# The effects of cadmium chloride on secondary metabolite production in *Vitis vinifera* cv. cell suspension cultures

**DOI:** 10.1186/0717-6287-47-47

**Published:** 2014-09-23

**Authors:** Emine Sema Cetin, Zehra Babalik, Filiz Hallac-Turk, Nilgun Gokturk-Baydar

**Affiliations:** Department of Horticulture, Faculty of Agriculture and Natural Science, Bozok University, 66200 Yozgat, Turkey; Fruit Research Station, Republic of Turkey Ministry of Food, Agriculture and Livestock, Egirdir, Isparta Turkey; Department of Horticulture, Faculty of Agriculture, Suleyman Demirel University, Isparta, Turkey; Department of Agricultural Biotechnology, Faculty of Agriculture, Suleyman Demirel University, Isparta, Turkey

**Keywords:** Cadmium chloride, Cell, Secondary metabolite

## Abstract

**Background:**

Plant secondary metabolites are possess several biological activities such as anti-mutagenic, anti-carcinogenic, anti-aging, etc. Cell suspension culture is one of the most effective systems to produce secondary metabolites. It is possible to increase the phenolic compounds and tocopherols by using cell suspensions. Studies on tocopherols production by cell suspension cultures are seldom and generally focused on seed oil plants. Although fresh grape, grape seed, pomace and grape seed oil had tocopherols, with our best knowledge, there is no research on tocopherol accumulation in the grape cell suspension cultures. In this study, it was aimed to determine the effects of cadmium chloride treatments on secondary metabolite production in cell suspension cultures of grapevine. Cell suspensions initiated from callus belonging to petiole tissue was used as a plant material. Cadmium chloride was applied to cell suspension cultures in different concentration (1.0 mM and 1.5 mM) to enhance secondary metabolite (total phenolics, total flavanols, total flavonols, *trans*-resveratrol, and α-, β-, γ- δ-tocopherols) production. Cells were harvested at two days intervals until the 6^th^ day of cultures. Amounts of total phenolics, total flavanols and total flavonols; *trans*-resveratrol and tocopherols (α-, β-, γ- and δ-tocopherols) and dry cell weights were determined in the harvested cells.

**Results:**

Phenolic contents were significantly affected by the sampling time and cadmium concentrations. The highest values of total phenolic (168.82 mg/100 g), total flavanol (15.94 mg/100 g), total flavonol (14.73 mg/100 g) and *trans*-resveratrol (490.76 μg/100 g) were found in cells treated with 1.0 mM CdCl_2_ and harvested at day 2. Contents of tocopherols in the cells cultured in the presence of 1.0 mM CdCl_2_ gradually increased during the culture period and the highest values of α, β and γ tocopherols (145.61, 25.52 and 18.56 μg/100 g) were detected in the cell cultures collected at day 6.

**Conclusions:**

As a conclusion, secondary metabolite contents were increased by cadmium chloride application and sampling time, while dry cell weights was reduced by cadmium chloride treatments.

## Background

Plant cell suspension culture is one of the most effective systems to produce secondary metabolites with high amount and purity. Using this system could ensure a continuous supply of uniform quality, specialized, natural components [1, 2] compared to traditional extraction methods. Unfortunately, the yield of the desired end-product is often too low to make this a commercially viable alternative to extraction from field grown plants. It is possible to increase the secondary metabolite accumulation in the cell culture by using some treatments such as light irradiation, UV, jasmonic acid, ozon, heavy metal, ethylene and sucrose [3, 4].

Plant cell culture is considered to be a potential means of producing valuable plant products in a factory setting. Among these products, food additives such as anthocyanins, shikonin compounds, safflower yellow, saffron and colorants are of high interest [5]. With the help of this technique, the presence of anthocyanins and *trans*-resveratrol were shown in *Vitis vinifera* suspension culture [6].

The phenolic compound family is huge and comprises a complex group of compounds varying from simple phenols to highly polymerised compounds. Polyphenols have been extensively studied and are reported to possess several biological activities. Numerous studies have focused on their anti-mutagenic chemopreventive and anti-carcinogenic activities [7–9].

Resveratrol (3, 4, 5-trihydroxystilbene), a natural polyphenol, is found in some plants that are used in human nutrition. Grape is the major source of resveratrol, and a significant amount can also be found in red wine. Several experimental studies have demonstrated biological properties of resveratrol, especially its anti-inflammatory, antioxidant, anti-platelet and antitumor effects [10]. Many reports have shown that resveratrol can prevent or slow the progression of a wide variety of illnesses, including cancer, cardiovascular disease and ischaemic injuries as well as enhance stress resistance and extend the lifespans of various organisms [11, 12]. Interest in this compound has been renewed in recent years, first from its identification as a chemopreventive agent for skin cancer, and subsequently from reports that it activates sirtuin deacetylases and extends the lifespans of lower organisms [13].

Tocopherols are another group of secondary metabolites produced in plant cell suspension cultures. Tocopherols as liposoluble, naturally occurring non-polar antioxidants comprising α (5, 7, 8-trimethyltocol), β (5, 8-dimethyltocol), γ (7, 8-dimethyltocol) and δ (8-methyltocol) tocopherols. Tocopherols appear to possess high antioxidant activity by donating the hydrogen of the hydroxyl group to the lipid peroxyl radical [14] and α-tocopherol is considered to be extremely important because of its vitamin E activity [15]. Tocopherols are ubiquitous, even if at different concentrations, in oil seeds, leaves and other green parts of higher plants [16].

Studies conducted on the production of tocopherols by cell suspension cultures are seldom and generally focused on seed oil plants [2, 17]. Although fresh grape [18], grape seed, pomace [19] and grape seed oil [20–22] had tocopherols in different amounts, with our best knowledge, there is no research on tocopherol accumulation in the grape cell suspension cultures.

Within this study, it was aimed to determine the effects of cadmium chloride (CdCl_2_) treatments on secondary metabolite production in the grape cell suspension cultures to be able to provide a preliminary reference for researchers who study or willing to study on this topic.

## Results and discussion

**Figure 1:**
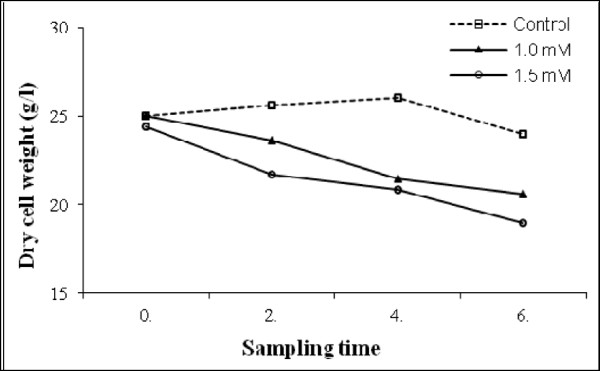
**Effects of cadmium chloride on the dry cell weight on cell suspension culture.**

In the CdCl_2_ treatments, cell suspension cultures were exposed to increasing concentrations of CdCl_2_ for up to 6 days. Changes in the dry cell weights were determined as g/L and data are showed in Figure 1. Dry cell weights varied depending on the CdCl_2_ concentrations and sampling time. It was determined that when the CdCl_2_ concentration increased and the sampling time extended, regular decreases were observed in the dry cell weights. 1.0 and 1.5 mM CdCl_2_ inhibited the growth of cells after 4 days and more dramatically after 6 days.

The data about the effects of cadmium chloride treatments on the phenolic contents of cell suspensions are presented in Table 1. Phenolic contents were significantly affected by the sampling time and cadmium concentrations (*p* ≤ 0.05). The highest values of total phenolic (168.80 mg/100 g), total flavanol (15.90 mg/100 g) and total flavonol (14.70 mg/100 g) were found in cells treated with 1.0 mM CdCl_2_ and harvested at day 2. Cell suspension cultures treated with 1.5 mM CdCl_2_ and sampled at days 4 and 6 gave the lowest amounts of total phenolics, total flavanols and total flavonols.

**Table 1:** **The effects of CdCl**_**2**_ **treatments on phenolic contents in Öküzgözü cell suspension cultures**

CdCl _2_ concentrations	Sampling time (day)	Total phenolics (mg/100 g)	Total flavanols (mg/100 g)	Total flavonols (mg/100 g)	***trans*** -resveratrol (μg/100 g)
**Control**	0.	102.84 e^*^	7.14 d	10.16 bc	138.12 e
	2.	128.32c	5.48 e	7.78 d	139.24 e
	4.	133.00 c	7.96 d	6.56 d	210.65 d
	6.	146.8 1b	9.42 c	6.65 d	216.42 d
**1.0 mM**	0.	100.64 ef	5.45 e	10.87 b	251.74 c
	2.	168.82 a	15.94 a	14.73 a	490.76 a
	4.	146.24 b	12.51 b	7.82 d	344.62 b
	6.	105.66 de	9.82 c	4.54 e	215.14 d
**1.5 mM**	0.	112.00 d	5.43 e	9.17 c	119.46 f
	2.	108.67 de	5.85 e	4.00 e	137.16 e
	4.	93.11 f	3.92 f	1.86 f	250.82 c
	6.	77.27 g	4.00 f	2.23 f	100.62 f

^*^Differences between means indicated by the same letters are not statistically significant (*p* ≤ 0.05).

In this study, the production of the *trans-*resveratrol in the cell cultures was significantly changed depending on the CdCl_2_ concentrations and sampling time (*p* ≤ 0.05). In the control group, *trans-*resveratrol showed a gradual increase during the sampling times and the highest value was detected in cell cultures harvested at day 6. In the cell cultures treated with 1.5 mM CdCl_2_, *trans-*-resveratrol significantly increased from day 0 to day 4, but it showed a sharp decrease at day 6. On the other hand the greatest *trans*-resveratrol content (490.76 μg/100 g) was detected in cell cultures treated with 1.0 mM CdCl_2_ and collected at the 2^nd^ day of the application followed by the cells cultured in the media containing 1.0 mM CdCl_2_ and harvested at day 4. But it subsequently exhibited a strong decrease in the cells collected at the 6^th^ day of the culture.

Tocopherol composition of the callus samples was affected significantly depending on the CdCl_2_ concentrations and the sampling times (P ≤ 0.05) as shown in Table 2. The effect was dose-dependent both in terms of time and CdCl_2_ concentration applied. In terms of tocopherols, α, β and γ-tocopherols were found at different concentrations depending on the treatments, while δ-tocopherol was not detected in the cell cultures of Öküzgözü. Contents of tocopherols in the cells cultured in the presence of 1.0 mM CdCl_2_ gradually increased during the culture period and the highest values of tocopherols were detected in the cell cultures collected at day 6. The analyses conducted in the present work revealed that these conditions are the most convenient conditions to stimulate the biochemical pathways leading to tocopherols. However, when the CdCl_2_ concentrations and the culture period increased, tocopherol contents of the cells decreased. Our data indicate that the lowest tocopherol concentrations were found in the cells treated 1.5 mM CdCl_2_ and sampled after 6 day.

**Table 2:** **The effects of CdCl**_**2**_ **treatments on tocopherol contents in Öküzgözü cell suspension cultures**

CdCl _2_ concentrations	Sampling time (day)	α- tocopherol (μg/100 g)	β- tocopherol (μg/100 g)	γ- tocopherol (μg/100 g)
**Control**	0.	118.52 c^*^	13.53 d	6.00 g
	2.	83.00 f	12.50 de	6.54 fg
	4.	82.41 f	17.00 c	12.46 b
	6.	86.12 f	11.55 e	11.34 c
**1.0 mM**	0.	97.18 e	11.00 e	4.00 h
	2.	113.00 cd	17.12 c	9.55 e
	4.	125.54 b	17.24 c	10.53 cd
	6.	145.61 a	25.52 a	18.56 a
**1.5 mM**	0.	102.58 e	17.00 c	7.14 f
	2.	109.55 d	21.24b	11.00 c
	4.	38.52 g	13.35 d	10.64 de
	6.	37.58 g	5.57f	2.20 i

^*^Differences between means indicated by the same letters are not statistically significant (*p* ≤ 0.05).

CdCl_2_ application inhibited the growth of cells. Because it is well known that when cadmium is excess in plants, it inhibits and disturbs various biochemical and physiological processes such as respiration, photosynthesis, cell elongation, plant-water relationships, nitrogen metabolism and mineral nutrition, resulting in poor growth, low biomass, cell death and inhibition of growth [23–25]. Similarly, treatment with Cd^2+^ at 0.05 mM appeared to inhibit cell division and induce either mitotic or total cell death in the sensitive tobacco cell subpopulation as reported by Kuthanova et al. [26].

Cell suspension cultures treated with 1.5 mM CdCl_2_ and sampled at days 4 and 6 gave the lowest amounts of total phenolics, total flavanols and total flavonols. These decreases can be resulted from the low cell weight in the presence of high CdCl_2_ concentrations and long exposure time. Our results confirmed that cytotoxic effects of cadmium in cells were concentration dependent and followed a distinct time course [27]. Similarly, Kuthanova et al. [26] found that treatment with Cd^2+^ in 1 mM concentration caused total and rapid cell death after 6 h, while application of 0.05 mM Cd^2+^ induced a marked decline of cell viability during the first 24 h of the cultivation in tobacco cells.

The accumulation of phenolic compounds represents a major key factor in the inducible defense mechanisms of plants through the phenylpropanoid pathway [28, 29]. The induction of the phenylpropanoid metabolism could also be achieved experimentally by treatments with elicitors or exposure to specific stress conditions [30, 31]. With respect to the high abundance of phenolic metabolites in plant tissues, regulation of phenylalanine ammonia lyase activity represents an important step in tolerance to stress factors [32]. Phenolic synthesis is recognized as a result of signalling processes initiated very quickly after injury, an attack of pathogens or elicitation [33, 34]. Kuthanova et al. [26] reported that 0.05 mM Cd^2+^ treated cells correlated with the stimulation of the activity of PAL, key enzyme in phenylpropanoid biosynthesis. They also found that Cd^2+^ treatment significantly stimulated PAL activity during the whole culture period which was 25 times higher on day 3 when compared to the control cells.

Cadmium is not an essential nutrient for plants and it is normally toxic [35, 36]. Its toxicity can promote altered metabolism [37] which can include the formation of reactive oxygene species (ROS) in plants under stress situations. Evidence confirmed that Cd stress induced the production of ROS such as superoxide, hydroxyl radicals (OH·) and hydrogen peroxide (H_2_O_2_) in plants [36]. However the interaction of Cd and antioxidative systems such as catalase, superoxide dismutase and glutathion reductase only recently have been studied in plant species [38, 39]. The degree of plant antioxidant enzyme activities under Cd stress was found in several distinct patterns, which varied according to Cd concentration, duration, the species and tissues [36]. On the other hand, very little is known about the the responses of grapevine cell cultures in terms of stress defence mechanisms under Cd [40].

## Conclusions

As a conclusion the results of our study showed that CdCl_2_ treatment can be used for enhancing phenolic compounds and tocopherols in grape cell cultures depending on the CdCl_2_ concentrations and exposure times. These increases might be explained by hypothesizing that Cd act as a stres factor on grape cell cultures which stimulate and alter the patways responsible for phenolics and tocopherol biyosynthesis. But high Cd concentrations and long exposure time had negative effects on cell viability and cell weight. When the treatment is used in high concentrations for long exposure durations, not only the cell division and cell viability but also the secondary metabolite accumulation decreases. To the best of our knowledge, this study reports the use of CdCl_2_ treatment to enhance phenolics and tocopherols in grape cell suspension culture for the first time. However, further investigations with various strategies for the phenolic and tocopherol contents should also be carried out in grape cell lines.

## Methods

In this research, callus tissues obtained from leaf petioles of Öküzgözü grape cultivar were used. Petioles were surface sterilised with commercial bleach (15%) for 15 min and rinsed three times with sterile distilled water. Petioles were then cut into 1 cm pieces and placed onto a solid B5 [41] culture medium with 30 g/L sucrose and 8 g/L bacto agar supplemented with 0.5 mg/L benzylaminopurine (BA), 0.5 mg/L indole acetic acid (IAA) and 2,4 dichlorophenoxyacetic acid (2,4-D). The pH was adjusted to 5.75. Explants were maintained at 25°C under dark conditions. Induced calli were subcultured on the same media in order to maintain sufficient stock cultures.

### Cell suspension cultures

Cell suspensions were initiated by inoculating fresh friable fragments of calli (2.5 g each) into 50 mL of liquid media in 250 mL Erlenmeyer flasks. Media were supplemented with macro elements (B5 medium), micro elements [42], vitamins [43], 0.1 mg/L naphtalen acetic acid, 0.2 mg/L kinetin, 250 mg/L casein hydrolizate and 20 g sucrose. Then, they were placed in a rotary shaker (100 rpm). The analyses were replicated three times.

### Cadmium chloride (CdCl_2_) treatment

At day 7, cell cultures were supplied with 1.0 and 1.5 mM CdCl_2_ dissolved in water. Control treatment did not contain CdCl_2_. Cells were harvested every 2d by filtration, rapidly washed, weighed and stored until day 6.

### Determination of dry cell weight

Growth kinetics were detrmined by obtaining the dry weight of the cell suspensions as g/L. Dry cell weights were determined after drying the biomass for 48 hours at 75°C.

### Determination of phenolic compounds

Phenolic compound extraction were carried out as previously described by Caponio et al. [44]. Total phenolic, total flavanols and total flavonol contents of the samples were determined spectrophotometrically using a PG Instruments T70 Plus Dual Beam Spectrophotometer (Arlington, MA, USA). Total phenolic contents were determined according to the Folin-Ciocalteu colorimetric method [45], calibrating against gallic acid standards and expressing the results as mg gallic acid equivalents (GAE) (mg/100 g). Total flavanol contents were determined according to the Arnous et al. [46], calibrating against catechin standards and expressing the results as mg catechin equivalents (CE) (mg/100 g). Total flavonol contents were determined according to Dai et al. [47], calibrating against rutin standards and expressing the results as mg rutin equivalents (RE) (mg/100 g). Data presented are average of three measurements.

HPLC analyses were performed on the HPLC system, Shimadzu Corp., Kyoto, Japan. The HPLC system was equipped with a pump (LC 10 AD), auto-sampler (SIL 10 AD), column oven (CTO 10A) and diode-array UV/VIS detector (DAD-λmax = 278). The separation was executed on a Agilent Eclipse XB C-18 (5 μm, 4.6 × 250 mm, Wallborn, Germany). The mobile phase was composed of acetic acid (2%) and methanol with the gradient elution system at a flow rate of 0.8 mL/min. For gradient elution, mobile phase A contained 3% acetic acid in water; solvent B contained methanol. The following gradient was used: 0–3 min, from 100% A to 95% A, 5% B; 3–20 min, from 95% A, 5% B to 80% A, 20% B; 20–30 min, from 80% A, 20% B to 75% A, 25% B; 30–40 min, from 75% A, 25% B to 70% A, 30% B; 40–50 min 70% A, 30% B to 60% A, 40% B; 50–55 min, 60% A, 40% B to 50% A, 50% B; 55–65 min, 50% A, 50% B to 100% B. The injection volume was 20 μL. Samples, standard solution of *trans*-resveratrol and mobile phases were filtered by a 0.45 μm pore size membrane filter (Millipore Co. Bedford, MA). The detection UV wavelength was set at 280 nm. The column temperature was set at 30°C. The compounds were quantified using Shimadzu CLASS-VP software. The contents of *trans*-resveratrol were determined on HPLC and expressing the results as μg/100 g. Data presented are average of three measurements.

### Determination of tocopherols

The extraction of tocopherols (α, β, γ and δ-tocopherol) were carried out as previously described by Caretto et al. [2]. Briefly, the method consisted of an alkaline hydrolysis (potassium hydroxide 60%) followed by extraction with *n*-hexane-ethyl acetate (9:1). Chromatography separation was performed by using a Beckman HPLC Analytical System. Luna Silica (250 × 4.6 mm) 5 μ column was used with heptane/tetrahydrofuran (95:5) as the mobile phase. RF-10AXL Floresan dedector was used to determine tocopherols. The tocopherol content was calculated as μg/100 g Fresh Cell Weight (FCW). Each experiment was carried out with at least three replicates.

### Statistical analysis

Data were subjected to analysis of variance with mean separation by Duncan’s multiple range test. Differences were considered statistically significant at the *p* ≤ 0.05 levels. Statistical analysis was performed using SPSS 16.0 for Windows.
